# Nanoscale measurement of trace element distributions in *Spartina alterniflora* root tissue during dormancy

**DOI:** 10.1038/srep40420

**Published:** 2017-01-18

**Authors:** Huan Feng, Yu Qian, J. Kirk Cochran, Qingzhi Zhu, Wen Hu, Hanfei Yan, Li Li, Xiaojing Huang, Yong S. Chu, Houjun Liu, Shinjae Yoo, Chang-Jun Liu

**Affiliations:** 1 Department of Earth and Environmental Studies, Montclair State University, Montclair, New Jersey 07043, USA; 2 School of Marine and Atmospheric Science, State University of New York, Stony Brook, NY 11794, USA; 3 National Synchrotron Light Source II, Brookhaven National Laboratory, Upton, New York 11973, USA; 4 College of Land and Environment, Shenyang Agricultural University, Shenyang 110866, PRC; 5 Computational Science Center, Brookhaven National Laboratory, Upton, New York 11973, USA; 6 Biological Sciences Department, Brookhaven National Laboratory, Upton, New York 11973, USA

## Abstract

This paper reports a nanometer-scale investigation of trace element (As, Ca, Cr, Cu, Fe, Mn, Ni, S and Zn) distributions in the root system *Spartina alterniflora* during dormancy. The sample was collected on a salt marsh island in Jamaica Bay, New York, in April 2015 and the root was cross-sectioned with 10 μm resolution. Synchrotron X-ray nanofluorescence was applied to map the trace element distributions in selected areas of the root epidermis and endodermis. The sampling resolution was 60 nm to increase the measurement accuracy and reduce the uncertainty. The results indicate that the elemental concentrations in the epidermis, outer endodermis and inner endodermis are significantly (*p* < 0.01) different. The root endodermis has relatively higher concentrations of these elements than the root epidermis. Furthermore, this high resolution measurement indicates that the elemental concentrations in the outer endodermis are significantly (*p* < 0.01) higher than those in the inner endodermis. These results suggest that the Casparian strip may play a role in governing the aplastic transport of these elements. Pearson correlation analysis on the average concentrations of each element in the selected areas shows that most of the elements are significantly (*p* < 0.05) correlated, which suggests that these elements may share the same transport pathways.

It has been reported that many plants acquire metals and other trace elements from the rhizosphere and regulate their uptake properties within the root system^1^,^2^,^3^,^4^,^5^,^6^. Thus, investigation of transport mechanisms that control the mobility and storage of these elements in the roots is important. As a survival strategy exhibited by many plant species, dormancy is a period of arrested plant growth in plant physiology, enabling the plants to survive conditions such as winter cold periods or dry seasons. Many plant species exhibit dormancy and have a biological clock that tells them when to slow activity and prepare soft tissues for a period of freezing temperatures or water shortage. The concentrations and distributions of elements in the plant root during dormancy are therefore in a quasi-steady state. This provides an opportunity to investigate the concentrations and distributions of these elements in the root system and the relationships among them. For over a decade, the role of Fe plaque in controlling metal mobility in the rhizosphere has been an issue of debate^7^,^8^,^9^. Investigation of the function of Fe plaque in metal transport processes can aid in understanding biogeochemical cycling of metals by plant roots, but traditional methods of measuring Fe plaque have technical limitations. For example, the traditional dithionite–citrate–bicarbonate (DCB) method that was used to extract Fe plaque in the root in previous studies^8^,^9^,^10^,^11^,^12^,^13^, only extracts Fe plaque on the root surface. This method is thus limited in extracting Fe from the entire root tissues and in mapping the distribution of Fe in the root cross-section. Therefore, it is difficult to fully understand the function of Fe plaque inside the root, especially around the Casparian strip, without high resolution measurements.

High-resolution nanoscale mapping techniques, such as the secondary ion mass spectrometry (SIMS) and synchrotron X-ray microfluorescence (μXRF), are useful for characterization of trace element concentrations and distributions in plants. Moore *et al*.^14^ summarized the application of NanoSIMS technique for determination of elemental distributions in plants^14^. Although NanoSIMS has a distinct advantage over synchrotron X-ray techniques in being able to detect light elements, the technique is not effective in studying heavy metals (e.g., Cd, Mn and Zn) due to its low ion yield^14^. The synchrotron μXRF technique has the advantage of overcoming this drawback of NanoSIMS and other traditional methods (e.g., DCB method) and has been successfully applied to studying metal concentrations and distributions in plants with micrometer scale resolution^15^,^16^,^17^,^18^,^19^,^20^,^21^,^22^,^23^.

Generally, a bulk chemistry approach gives information that averages over different components of the plants. Unlike conventional wet chemical analyses, the synchrotron-based techniques have demonstrated advantages in sample preparation and measurement. Synchrotron radiation measurement gives detailed views of the accumulation of metals and their chemistry on individual spots or within small areas in the plants including roots, stems, and leaves^18^,^19^,^20^,^21^,^22^,^23^. For example, a multilayer Laue lens (MLL)-based scanning hard-X-ray microscope^24^ has been developed at the Hard X-ray Nanoprobe (HXN) beamline at National Synchrotron Light Source II (NSLS-II). This beamline can provide an ultra-high spatial resolution measurement down to about a 10 nm level for various scientific applications^25^,^26^,^27^. The unique feature of this high detection sensitivity and resolution measurement is to make each pixel size small enough to focus on a specific small area in the root tissue during the measurement. This feature can lead to a better understanding of the transport and distributions of the trace elements in the plants. Thus, synchrotron X-ray nanoprobe measurements can provide new insights into the mechanisms taking place in the plants during the course of metal uptake and transport at levels where interactions can be understood. Here, we apply the synchrotron X-ray nanoprobe technique to investigate trace element distributions in the root tissue of the salt marsh cord grass, *Spartina alterniflora* from Jamaica Bay, New York. The purpose of this study is to improve our current knowledge of transport and accumulation of trace elements in the root system in order to better understand the ecological function of the salt marsh ecosystems as habitats and nurseries for marine life.

## Results

### Concentrations and spatial distributions of trace elements in the root system

**Figure 1:**
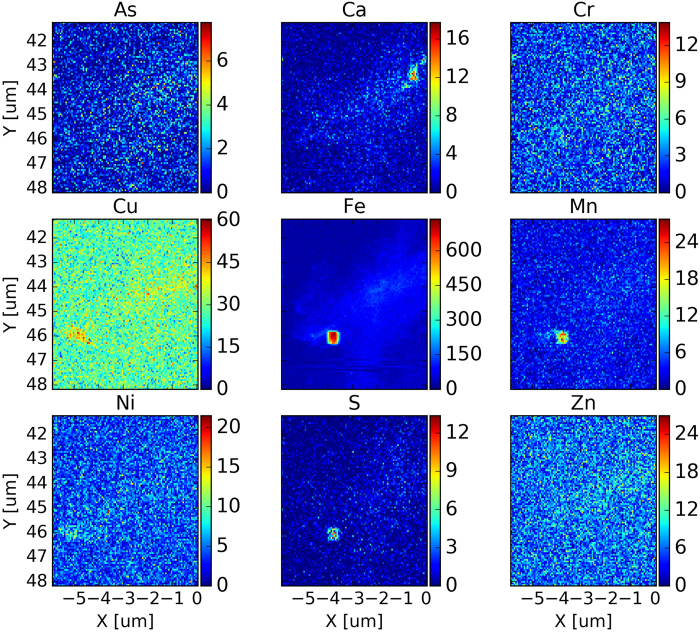
High-resolution synchrotron X-ray nanofluorescence (nano-XRF) images (pixel size = 60 nm) showing distributions of As, Ca, Cr, Cu, Fe, Mn, Ni, S and Zn in *Spartina alterniflora* root epidermis (Area S2493). This nanometer scale mapping is able to provide more accurate information of the elemental distributions in the tissue than that from micrometer scale mapping and, hence, reduce the uncertainty. The color bars show the concentration in the units of counts per second (cps). The dimension of the area is approximately 6 μm × 6 μm.

**Figure 2:**
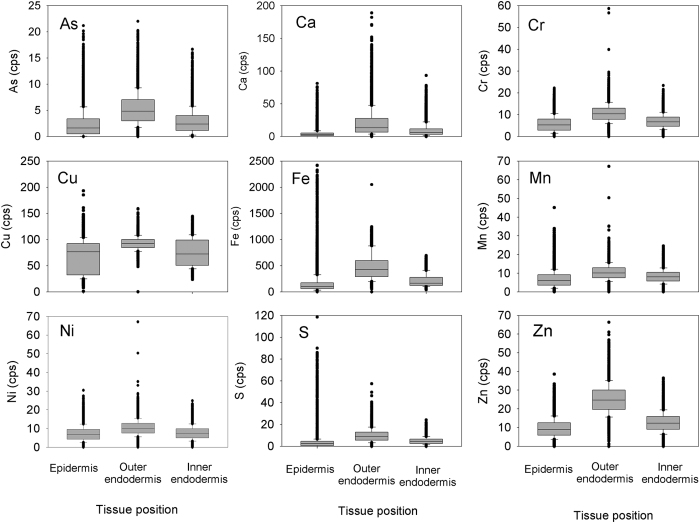
Box plot of As, Ca, Cr, Cu, Fe, Mn, Ni, S and Zn concentrations and variations in the root epidermis and the outer and inner endodermis around the Casparian strip. The results indicate that these elements in outer endodermis have relatively higher concentrations than that in the other layers, suggesting that the Casparian strip plays a role in the elemental transport in the root system.

Examples of the distributions of As, Ca, Cr, Cu, Fe, Mn, Ni, S and Zn in each selected area in the epidermis (Areas S2493, S2496 and S2498) and the endodermis (Areas S2504, S2505, S2416 and S2418) are shown in Fig. 1 and Supplemental Information Figures S1–S6. Statistical analyses of the elemental concentrations in each selected area are summarized in Table 1. The nanometer scale high-resolution mapping indicates localized high concentration areas (“hot spots”) of these elements in the root tissue. For example, very high Fe concentration was found in several areas in both the epidermis and endodermis. Sulfur (S) showed a distribution pattern very similar to Fe in both epidermis and endodermis. The result suggests that Fe and S may form Fe-S compounds or precipitates and be deposited in the root tissue. The distribution pattern of Ca in the root epidermis is also very similar to that of Fe. Both Cr and Ni were found relatively evenly distributed in the epidermis and the endodermis. Other elements (As, Cu, Mn and Zn) showed patchiness, with areas of high concentrations in both epidermis and endodermis (Figs 1 and S1–S6). The results suggest that the distributions of these elements in the root tissue are not uniform. In further data analysis, the seven areas were clustered according to their locations in the root, i.e., epidermis (S2493, S2496 and S2498), outer endodermis (S2504 and S2516), and inner endodermis (S2505 and S2518). As shown in Fig. 2, the median concentrations of As, Ca, Cr, Cu, Fe, Mn, Ni, S and Zn in the endodermis were generally higher than those in the epidermis although the concentrations of these elements in each selected area of the root epidermis and endodermis vary widely. Furthermore, the median concentrations of these trace elements in the outer endodermis are generally higher than those in the inner endodermis (Fig. 2). The results show a significant (p < 0.01) difference, based on a two-sample t-test, in the elemental concentrations between the inner endodermis and outer endodermis (Table 2).

### Statistical analyses of trace elements in the root system and relationship with Fe

**Figure 3:**
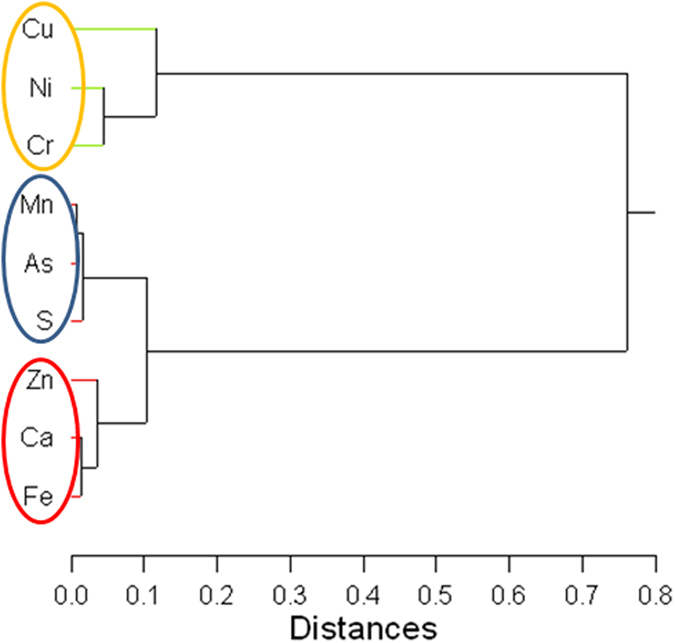
Results of the hierarchical clustering analysis showing the relevant associations among the elements measured. Three clusters are evident: 1) Cu, Ni Cr; 2) Mn, As and S; and 3) Zn, Ca and Fe. The joining algorithm is the Ward minimum variance method and the distance metrics are based on the Pearson correlation coefficient.

A one-way analysis of variables (ANOVA) was performed to examine the differences in concentrations among the epidermis, outer endodermis and inner endodermis. The results show statistically significant (*p* < 0.01) differences in the elemental concentrations among these layers (Table 3). Based on the mean concentration, which is an average of the concentrations in each selected area in the root tissue (Table 1), Pearson liner correlation analysis was performed to examine the relationship between the trace elements (As, Ca, Cr, Cu, Fe, Mn, Ni, S and Zn). The results show that most of these metals are significantly correlated at the 5% two-tailed significance level (Table 4). Hierarchical cluster analysis, which is also based the average concentration of each individual area in the root tissue (Table 1), shows that these elements (As, Ca, Cr, Cu, Fe, Mn, Ni, S and Zn) can be divided into three clusters, i.e., Cu, Ni and Cr; Mn, As and S; and Zn, Ca and Fe (Fig. 3).

## Discussion

Wetland plants need essential nutrients for their growth. Commonly, they uptake and transport the nutrients from root epidermis to vascular bundle and then to stem and leaf^4^,^5^,^28^,^29^. Therefore, the root system is an active site for absorption of nutrients from the rhizosphere^30^,^31^,^32^. Trace elements such as Fe, Mn, Cu, Ni and Zn are essential micronutrients for plant growth, while As and Cr are not. Thus, transport and accumulations of these metals in the root tissues can be different^4^,^5^,^33^. Because non-essential elements, such as As and Cr, are not necessary for plant growth^33^,^34^, they are usually not actively taken up from rhizosphere and transported within the plant tissues.

**Figure 4:**
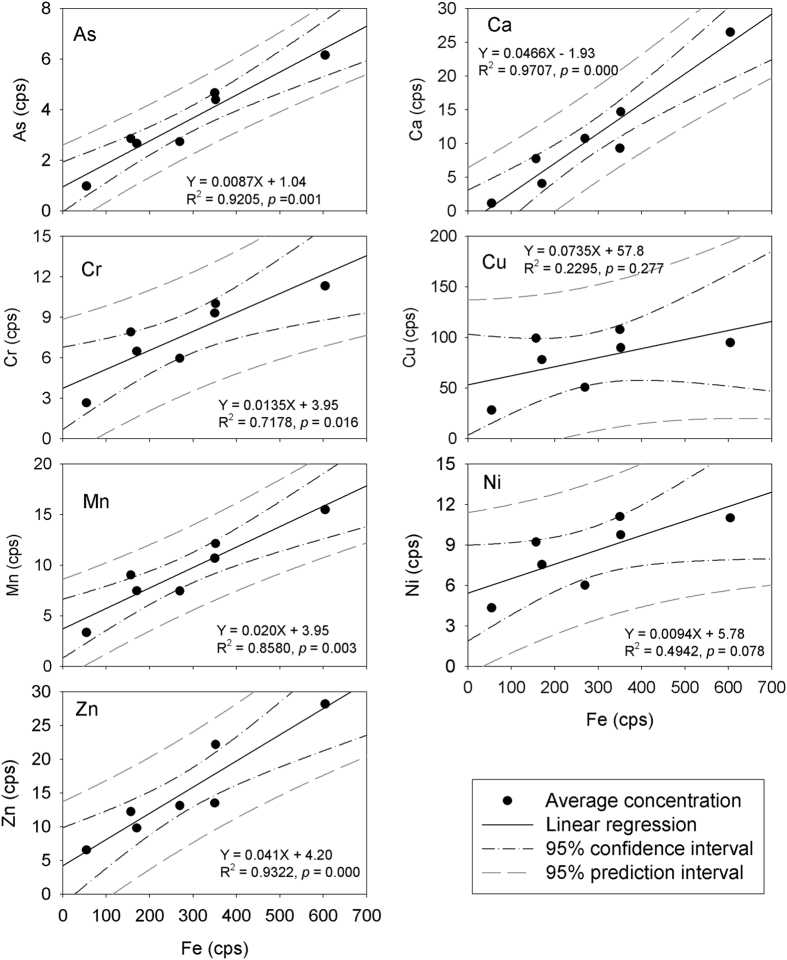
A significant linear correlation between Fe and S suggests formation of iron sulfide and/or Fe-S clusters in the *Spartina alterniflora* root.

Iron is an example of a micronutrient element for plants, and as a consequence, should be actively taken up. Previous synchrotron X-ray measurement indicated that Fe could be present in the root tissue as Fe nanoparticles, dominated by Fe^3+^ 19,20. More specifically, some prior studies reported that Fe accumulations did not occur to any extent within the outer epidermal wall or cortex of *Spartina alterniflora* roots^35^. The penetration depth of Fe into the roots was only up to 15–17 μm, a depth approximating the thickness of an epidermal cell^36^. In contrast, however, other studies reported that Fe was found in the cells of the root rhizodermis, exodermis and endodermis, including the Casparian strip^37^. Our high resolution synchrotron nano-XRF measurements show that Fe not only deposits in the epidermis, but also has considerable deposition around the Casparian strip in the endodermis. Previous studies also suggested that Fe plaque could serve as a barrier preventing metals from entering plant roots^37^,^38^,^39^, while others suggested that Fe plaque should not be the main barrier^10^. Our results show significant (*p* < 0.05) correlations between the metals (As, Ca, Cr, Mn and Zn) and Fe in both epidermal and endodermal root tissue (Fig. 4), suggesting that these trace elements are scavenged by Fe. In contrast, Cu and Ni show no significant (*p* > 0.05) correlations with Fe (Fig. 4), implying that scavenging by Fe is not a main barrier to these elements during their transport.

**Figure 5:**
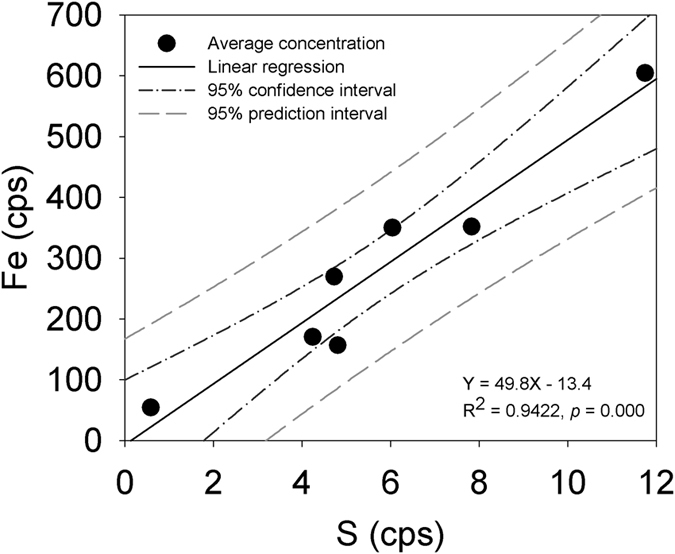
Results of Pearson linear regression analyses of As, Ca, Cr, Cu, Mn, Ni and Zn with Fe in the *Spartina alterniflora* root epidermis and endodermis. The significance levels of the correlation are indicated by the *p* values. As, Ca, Cr, Mn and Zn show significant correlation with Fe (95% confidence level), while Cu and Ni show no significant correlation with Fe (95% confidence level). The results suggest that Fe can scavenge the metals and impact their transport in the root system.

Early studies found that the rhizosphere is a favorable environment for microbial communities^40^,^41^,^42^,^43^. As a consequence of early diagenetic reactions in the salt marsh system, a sulfide phase may assist in explaining the observed metal transport and distributions. Bacterial sulfate reduction can result in elevated hydrogen sulfide concentrations in marsh peat pore water, causing the precipitation of metal sulfides (e.g., FeS). Indeed, the co-existence of Fe and S in the root tissue (Fig. 1) and the significant (*p* < 0.01) correlation between Fe and S (Fig. 5) suggest the possible presence of an Fe-S phase.

**Figure 6:**
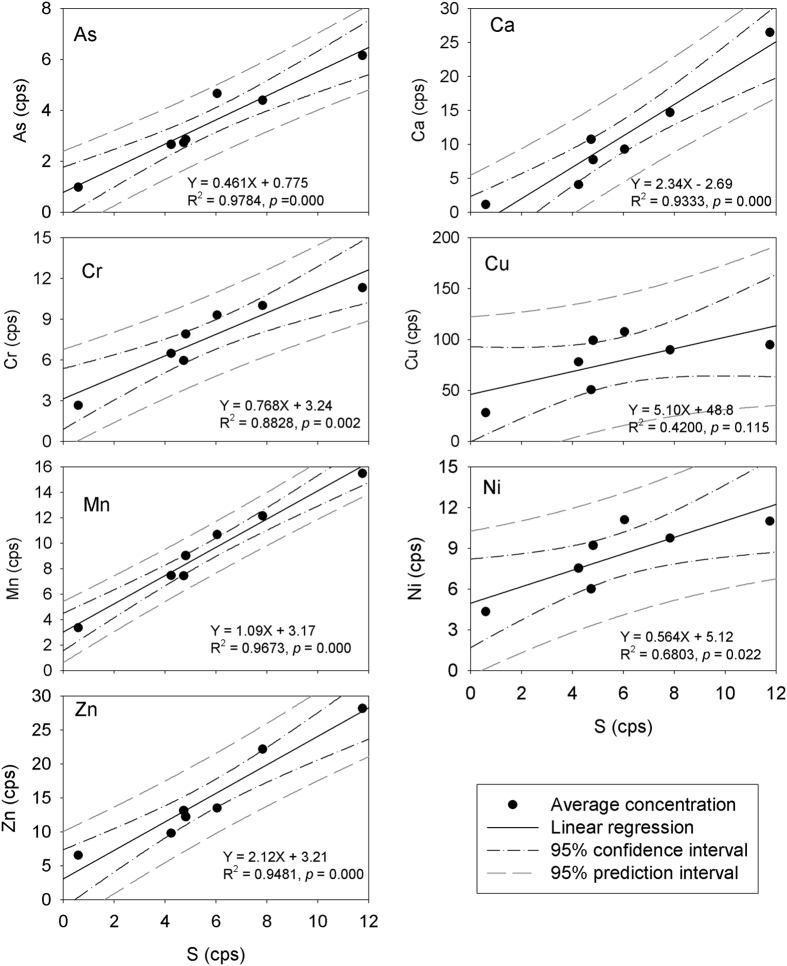
Results of Pearson linear regression analyses of As, Ca, Cr, Mn, Ni and Zn with S in the *Spartina alterniflora* root. The significance levels of the correlation are indicated by *p* values. All the elements except Cu show significant correlation with S at the 95% confidence level. The relationships suggest that S may precipitate or cluster other elements during the transport in the root system.

Iron-sulfur (Fe–S) clusters built of iron and acid-labile sulfide are ubiquitous/central prosthetic groups required to sustain fundamental life processes. These include photosynthesis, respiration and nitrogen fixation^44^. The main function of Fe–S proteins is electron transfer through the redox cycling of Fe (Fe^2+^ or Fe^3+^). It has been reported that Fe–S cluster biosynthesis involves complex biochemical pathways, and the chemical properties of the Fe–S cluster enable the associated proteins to be involved in metabolic processes^44^,^45^. Therefore, FeS and/or Fe-S clusters could be in part responsible for the strong correlations of these elements such as As, Ca, Cr, Mn, Ni and Zn with S (Fig. 6). Although Cu does not show a significant (*p* < 0.05) correlation with S (Fig. 6), it does show significant (*p* < 0.05) correlations with Mn (Table 4).

Indeed, the present study shows that all the elements (As, Ca, Cr, Cu, Fe, Ni, S and Zn) are significantly (*p* < 0.05) correlated with Mn (Table 4). Like Fe, Mn is an important essential micronutrient for wetland plants to create the organic compounds that make up plant tissue or drive growth processes. It is a structural component of the chloroplasts where photosynthesis occurs. Therefore, Mn is irreplaceable and necessary for plant growth and reproduction. Manganese (Mn) can be present in wetland plants as Mn oxides or Fe-Mn plaque (Fe-Mn oxides), which can scavenge other metals by adsorption or co-precipitation during transport.

To further examine the close relationships among the elements (As, Ca, Cr, Cu, Fe, Mn, Ni, S and Zn) in the root tissue (i.e., epidermis and endodermis), hierarchical cluster analysis was performed on the data. As shown in Fig. 3, there are three main clusters, i.e., Cu, Ni and Cr; Mn, As and S; and Zn, Ca and Fe. Elements in the same cluster correlate with each other better than with the elements in other clusters, and thus may have the same transport mechanisms or share similar transporting agents. Although the investigation of the exact mechanisms is beyond the scope of this study, the results indicate that the transport of the measured trace elements within the plant tissues are via different pathways or transport proteins. Some elements may share the same transport pathway or be part of a similar transport protein in the root^46^,^47^. As well, expression of uptake and transport of these elements by the root may be different.

## Conclusions

This investigation applies synchrotron nano-XRF technique to studying trace element concentrations and distributions in *Spartina alterniflora* root system with a resolution of nanometers. High concentrations of trace elements (As, Ca, Cr, Cu, Fe, Mn, Ni, S and Zn) were found in localized areas in the root epidermis and endodermis. Despite the fact that metal concentrations vary widely in the root tissue, statistical analysis indicates that the outer endodermis has relatively higher concentrations of these elements than the epidermis and inner endodermis. The results imply that the Casparian strip may play a role in elemental transport. Significant (*p* < 0.01) correlation between Fe and S suggest the formation of an Fe-S precipitate or Fe-S cluster in the root tissue. As well, significant (*p* < 0.01) correlations of As, Ca, Cr, Mn, Zn and (Ni) with Fe and S in the epidermis and the endodermis suggest scavenging by Fe plaque, sulfide precipitation and/or Fe-S cluster complexation play an important (perhaps combined) role in metal transport in the root tissue. Linear regression analysis shows that As, Ca, Cr, Cu, Fe, Ni, S and Zn are significantly (*p* < 0.05) correlated with Mn, suggesting that the divalent metal (e.g., Mn^2+^) may be also responsible for the metal transport.

## Materials and Methods

### Sample collection and preparation

Field work for the sample collection was conducted in April 2015 in Elders Point East marsh in Jamaica Bay, Long Island, New York. Elders Point East was heavily degraded and was restored in 2006–2007 by the US Army Corps of Engineers. The restoration involved the emplacement of dredged sand to elevate the marsh surface and the planting of 700,000 salt marsh cord grass (*S. alterniflora*) in the low marsh and salt hay (*S. patens*) in the high marsh zones. A sample of *S. alterniflora* was collected with the associated peat at a site (40°38′10.208″N, 73°50′44.59″W) using stainless steel spades, placed into large plastic containers and then transported to Stony Brook University for further treatment. Soils were carefully removed from the roots by hand and the trace residual soils on the roots were carefully rinsed off with small amounts (<20 ml) of deionized water.

The sample was further treated at Brookhaven National Laboratory. A sample of fresh root with a diameter of ~1 mm was selected for synchrotron nanofluorescence (nano-XRF) measurement. The root was suspended in an optimal-cutting-temperature (OCT) compound (Surgipath Medical Industries, Surgipath, Canada), which is used to embed tissue samples prior to frozen sectioning on a microtome-cryostat and does not infiltrate the specimen. The sample was rapidly cooled to −20 °C, and once the OCT compound solidified, a cryotome (Cryostat CM1950, Leica Microsystems) was used to cut a 10 μm thin section. The thin section of the root sample was mounted on a silicon nitride membrane window (Norcada Inc., Edmonton, Canada), with an area of 1 mm × 0.5 mm for sample placement. The sample was then fixed on an aluminum holder for synchrotron nano-XRF analysis. Between the preparation of the thin section and the synchrotron nano-XRF analysis, the mounted sample was kept in a desiccator at the Hard X-ray Nanoprobe (HXN) Beamline at the National Synchrotron Light Source II (NSLS-II) of the Brookhaven National Laboratory (Upton, NY).

### Synchrotron X-ray nanofluorescence (nano-XRF) measurement

**Figure 7:**
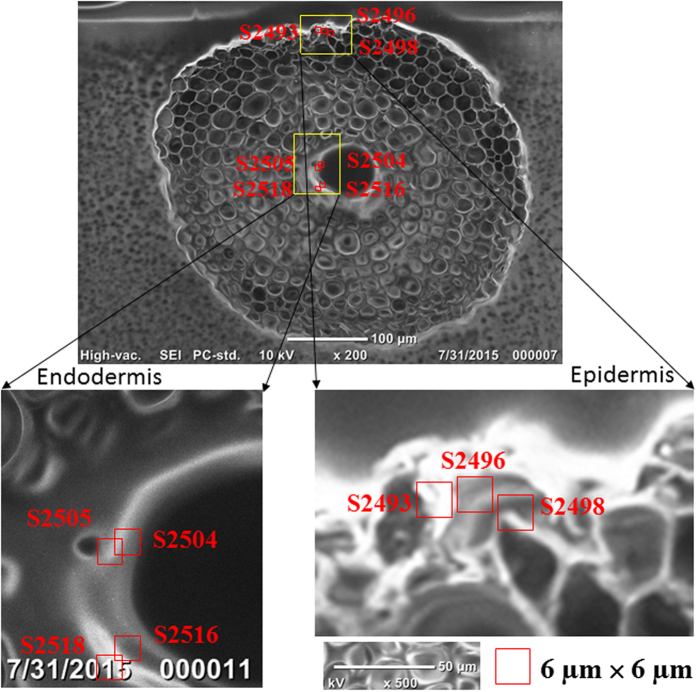
Optical images showing the cross-sections of *Spartina alterniflora* root structure. The framed areas are selected for synchrotron XRF measurement and extracted for data analysis. The thickness of the cross-sections is 10 μm. Among the selected areas, S2493, S2496 and S2498 are located in the epidermis of the root, S2505 and S2518 are in the outer endodermis, and S2504 and S2516 are in the inner endodermis.

Concentrations and distributions of the trace elements in *S. alterniflora* root were investigated at the Hard X-ray Nanoprobe (HXN) Beamline at NSLS-II. The HXN beamline provides a scanning X-ray microscopy capability with multimodality imaging including fluorescence and differential phase contrast^27^,^48^ at a 15 nm spatial resolution. In this study, the nano-XRF measurement was carried out at 12 keV using a HXN X-ray microscope^24^ equipped with a pair of multilayer Laue lenses. Seven small areas with a dimension of approximately 6 μm × 6 μm were selected in the root epidermis and endodermis around the Casparian strip (Fig. 7). Specifically, three areas (S2493, S2496 and S2498) were in the epidermis, two areas (Areas S2505 and S2418) were in the outer endodermis and two areas (Areas S2504 and S2416) were in the inner endodermis (Fig. 7). In order to examine the function of Casparian strip in the elemental transport, there were some overlaps in the areas chosen in the outer and inner endodermis. Fluorescence imaging scans, via continuous fly-scan^49^, were performed with a sampling resolution of 60 nm per pixel and dwell time of either 0.15 or 0.25 second per pixel, depending on the signal strength. The fitting of the X-ray fluorescence data was accomplished using PyXRF, an X-ray fluorescence analysis package developed at the NSLS-II.

### Data analysis

Extraction of the data matrix was made possible by Matlab (MathWorks) and the data matrix of each element was then converted to a linear dataset for further analysis. Statistical analysis was performed using Systat (Systat Software, Inc.). In hierarchical cluster analysis, the joining algorithm used to amalgamate clusters was Ward’s method, which assesses the relationship between each cluster by calculating the total sum of squared deviations from the mean of a cluster, and the metric for measuring distance for the raw and standardized data was the Pearson correlation coefficient.

## Additional Information

**How to cite this article**: Feng, H. *et al*. Nanoscale measurement of trace element distributions in *Spartina alterniflora* root tissue during dormancy. *Sci. Rep.* **7**, 40420; doi: 10.1038/srep40420 (2017).

**Publisher's note:** Springer Nature remains neutral with regard to jurisdictional claims in published maps and institutional affiliations.

## Supplementary Material

Supplementary Information

## Figures and Tables

**Table 1:** Statistical results of the concentrations of trace elements in each selected area in *S. alterniflora* root: median, mean, standard deviation and variation range.

Scan #	Statistics	As	Ca	Cr	Cu	Fe	Mn	Ni	S	Zn
S2493	n	10201	10201	10201	10201	10201	10201	10201	10200	10201
S2493	min	0.000	0.000	0.000	0.783	1.07	0.000	0.000	0.000	0.000
S2493	max	7.60	17.4	13.6	59.2	723.4	27.3	21.1	13.1	26.4
S2493	median	0.69	0.86	2.46	27.8	44.9	3.04	4.08	0.10	6.28
S2493	mean	0.98	1.15	2.66	28.2	54.3	3.37	4.34	0.59	6.57
S2493	s.d.	1.04	1.41	1.83	6.14	46.95	2.19	2.36	0.89	3.29
S2496	n	10201	10201	10201	10201	10201	10201	10201	10201	10201
S2496	min	0.000	0.000	0.019	54.4	34.0	0.323	0.137	0.000	0.000
S2496	max	21.2	74.4	22.2	253	1815	33.0	30.4	29.1	38.6
S2496	median	2.80	5.56	7.73	97.0	190	8.88	9.40	4.28	12.0
S2496	mean	3.42	6.68	7.94	97.2	220	9.21	9.69	4.62	12.2
S2496	s.d.	2.83	5.25	3.15	12.3	135	3.67	3.66	2.65	5.08
S2498	n	10201	10201	10201	10201	10201	10201	10201	10201	10201
S2498	min	0.000	0.000	0.000	44.7	35.0	0.000	0.000	0.000	0.000
S2498	max	20.2	81.1	21.6	124	2421	45.0	27.4	119	28.0
S2498	median	2.06	3.37	6.22	77.6	103	7.05	7.30	3.25	9.54
S2498	mean	2.67	4.07	6.48	78.0	170	7.47	7.54	4.24	9.80
S2498	s.d.	2.47	4.24	2.92	10.3	226	3.48	3.12	6.51	4.40
S2504	n	10201	10201	10201	10201	10201	10201	10201	10201	10201
S2504	min	0.000	0.000	0.000	54.1	41.6	0.102	0.308	0.000	0.000
S2504	max	16.7	75.5	23.4	144	450	24.5	24.8	21.2	33.6
S2504	median	2.31	5.26	7.68	99.1	136	8.80	8.93	4.46	11.9
S2504	mean	2.85	7.73	7.91	99.3	157	9.04	9.22	4.81	12.2
S2504	s.d.	2.44	8.04	3.10	11.6	65.4	3.42	3.43	2.60	5.01
S2505	n	10201	10201	10201	10201	10201	10201	10201	10201	10201
S2505	min	0.000	0.000	0.000	48.1	101	0.181	0.000	0.000	1.189
S2505	max	16.9	135	58.7	159	1129	32.3	50.3	34.2	51.9
S2505	median	4.05	9.04	9.76	89.6	341	11.9	9.51	7.24	21.9
S2505	mean	4.40	14.70	10.0	90.0	352	12.1	9.76	7.83	22.2
S2505	s.d.	2.52	15.87	3.67	11.8	133	4.13	3.82	4.36	6.65
S2516	n	10201	10201	10201	10201	10201	10201	10201	10201	10201
S2516	min	0.000	0.000	0.000	23.3	39.6	0.000	0.000	0.000	0.000
S2516	max	14.0	93.3	19.0	94.1	693	22.8	21.6	24.0	36.4
S2516	median	2.43	7.00	5.69	50.5	242	7.15	5.74	3.95	12.8
S2516	mean	2.74	10.75	5.96	50.8	270	7.45	6.02	4.73	13.1
	s.d.	1.99	10.44	2.81	8.31	140	3.23	2.89	3.53	5.08
S2518	n	9633	9633	9633	9633	9633	9633	9633	9633	9633
S2518	min	0.000	0.000	0.000	0.000	0.000	0.000	0.000	0.000	0.000
S2518	max	22.0	203	56.7	337	2048	67.3	67.1	57.4	66.3
S2518	median	5.82	20.4	11.1	94.7	576	15.2	10.7	11.0	28.0
S2518	mean	6.16	26.5	11.3	94.9	604	15.5	11.0	11.8	28.2
S2518	s.d.	3.21	21.7	3.9	12.3	266	5.15	4.00	6.22	7.50

The concentration unit is in counts per second (cps). S2493, S2496 and S2498 are measured in the epidermis, and S2504, S2505, S2516 and S2518 are in the endodermis.

(Abbreviation: n = number of data points, min = minimum, max = maximum, and s.d. = standard deviation).

**Table 2:** Results of two-sample t-test compare concentration differences between inner endodermis and outer endodermis for the indicated metals.

Element	Tissue component	Number of data	Mean	Standard deviation	*p-*value
As	Inner endodermis	20402	2.79	2.23	0.000
	Outer endodermis	19834	5.25	3.01	
Ca	Inner endodermis	20402	9.24	9.44	0.000
	Outer endodermis	19834	20.43	19.82	
Cr	Inner endodermis	20402	6.93	3.12	0.000
	Outer endodermis	19834	10.65	3.86	
Cu	Inner endodermis	20402	75.0	26.3	0.000
	Outer endodermis	19834	92.4	12.3	
Fe	Inner endodermis	20402	213	123	0.000
	Outer endodermis	19834	475	244	
Mn	Inner endodermis	20402	8.24	3.42	0.000
	Outer endodermis	19834	13.77	4.95	
Ni	Inner endodermis	20402	7.62	3.55	0.000
	Outer endodermis	19834	10.36	3.96	
S	Inner endodermis	20402	4.77	3.10	0.000
	Outer endodermis	19834	9.74	5.69	
Zn	Inner endodermis	20402	12.7	5.1	0.000
	Outer endodermis	19834	25.1	7.7	

**Table 3:** Summary of one-way analysis of variance (ANOVA) test results.

Univariate F Tests					
Source	Type III SS	Degree of freedom	Mean squares	F-ratio	*p-value*
As	99890	2	49945	7191	0.000
Error	510072	73436	6.95		
Ca	3214924	2	1607462	11387	0.000
Error	10366966	73436	141		
Cr	280137	2	140068	11064	0.000
Error	929670	73436	12.7		
Cu	5914697	2	2957349	4329	0.000
Error	50166621	73436	683		
Fe	1263000000	2	631700000	18599	0.000
Error	2494000000	73436	33965		
Mn	594723	2	297362	16878	0.000
Error	1293809	73436	17.6		
Ni	115348	2	57674	3909	0.000
Error	1083412	73436	14.8		
S	516332	2	258166	12720	0.000
Error	1490425	73436	20.3		
Zn	3009492	2	1504746	43488	0.000
Error	2540970	73436	34.6		

According to the root areas analyzed, data are grouped into the root epidermis, inner endodermis and outer endodermis. There are significant differences in metal concentrations among these different root tissue layers.

**Table 4:** The result of Pearson correlation matrix shows the relationships of the area-averaged trace metal concentrations, which are based on the seven individual areas (n = 7) in the root epidermis and endodermis.

	As	Ca	Cr	Cu	Fe	Mn	Ni	S	Zn
As	1.000								
Ca	0.941	1.000							
Cr	0.957	0.842	1.000						
Cu	0.700	0.469	0.851	1.000					
Fe	0.959	0.985	0.847	0.479	1.000				
Mn	0.991	0.923	0.983	0.758	0.926	1.000			
Ni	0.872	0.692	0.955	0.950	0.703	0.909	1.000		
S	0.989	0.966	0.940	0.648	0.971	0.984	0.825	1.000	
Zn	0.961	0.974	0.897	0.539	0.966	0.954	0.757	0.974	1.000

The underlined data indicate no significant correlation at 95% confidence level.
