# Supplementary material for: Nanoscale measurement of trace element distributions in Spartina alterniflora root tissue during dormancy
Source: Sci Rep. 2017 Jan 18;7:40420. doi: 10.1038/srep40420 (PMC5241796; doi:10.1038/srep40420)
Supplement: Supplementary Information [file srep40420-s1.pdf]

Nanoscale measurement of trace element distributions in *Spartina alterniflora* root tissue during dormancy

Huan Feng [1*], Yu Qian [1‡], J. Kirk Cochran [2], Qingzhi Zhu [2], Wen Hu [3], Hanfei Yan [3], Li Li [3], Xiaojing Huang [3], Yong S. Chu [3], Houjun Liu [4], Shinjae Yoo [5] and Chang-Jun Liu [6]

1. Department of Earth and Environmental Studies, Montclair State University, Montclair, New Jersey 07043, USA
2. School of Marine and Atmospheric Science, State University of New York, Stony Brook, NY 11794, USA
3. National Synchrotron Light Source II, Brookhaven National Laboratory, Upton, New York 11973, USA
4. College of Land and Environment, Shenyang Agricultural University, Shenyang 110866, PRC
5. Computational Science Center, Brookhaven National Laboratory, Upton, New York 11973, USA
6. Biological Sciences Department, Brookhaven National Laboratory, Upton, New York 11973, USA

[*] Correspondence e-mail: fengh@mail.montclair.edu

[‡] Current Address: School of Ecology and Environmental Sciences, Yunnan University, Kunming, Yunnan 650091, People's Republic of China.

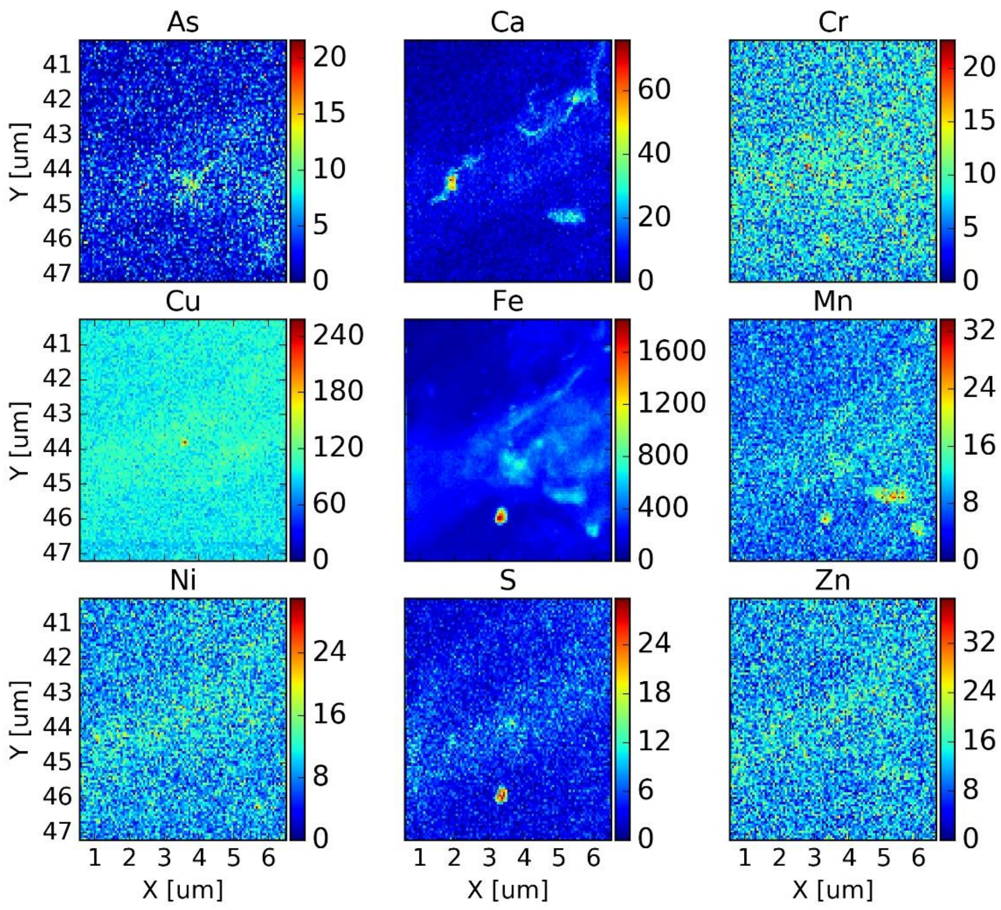

Figure S1. High resolution images (pixel size = 60 nm) from synchrotron X-ray nanofluorescence (nano-XRF) measurement showing distributions of As, Ca, Cr, Cu, Fe, Mn, Ni, S and Zn in *Spartina alterniflora* root epidermis (Area S2496). This nanometer scale mapping is able to provide more accurate information of the elemental distributions in the tissue than that from the micrometer scale measurement and, hence, reduce the uncertainty. The color bars show the concentrations in the units of counts per second (cps). The dimension of the area is approximately 6 µm × 6 µm.

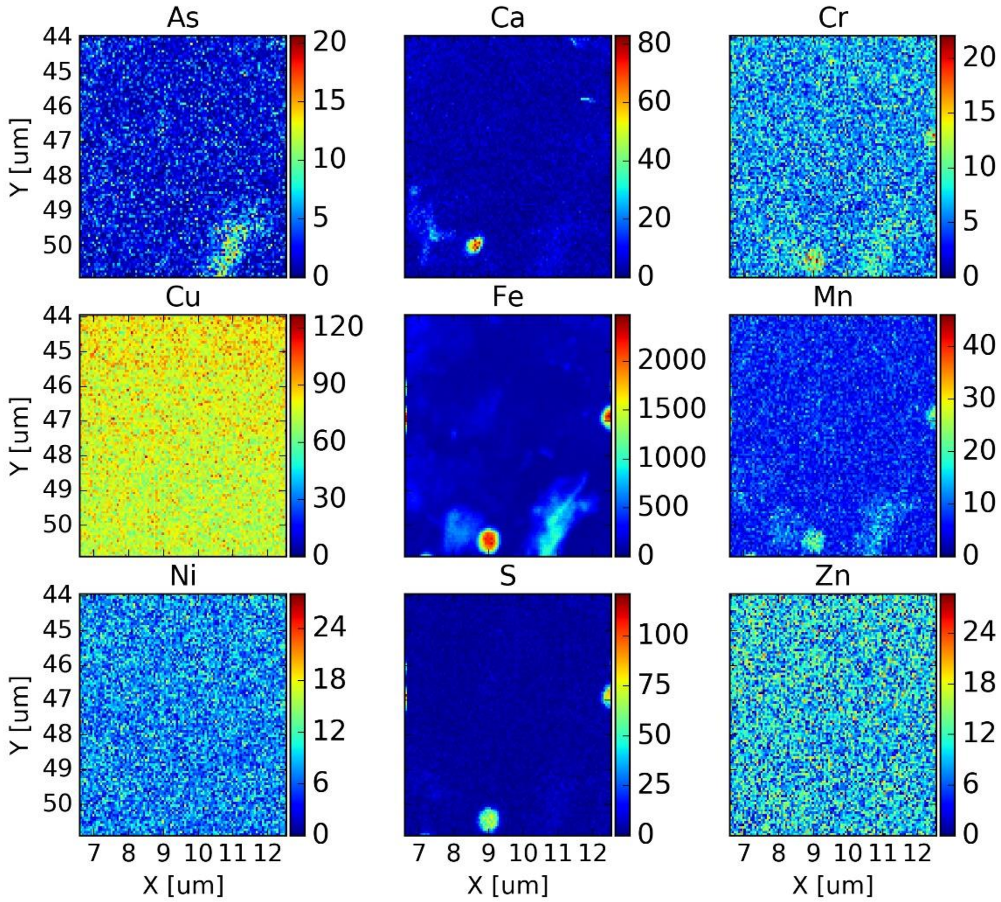

Figure S2. High resolution images (pixel size = 60 nm) from synchrotron X-ray nanofluorescence (nano-XRF) measurement showing distributions of As, Ca, Cr, Cu, Fe, Mn, Ni, S and Zn in *Spartina alterniflora* root epidermis (Area S2498). This nanometer scale mapping is able to provide more accurate information of the elemental distributions in the tissue than that from the micrometer scale measurement and, hence, reduce the uncertainty. The color bars show the concentrations in the units of counts per second (cps). The dimension of the area is approximately 6 µm × 6 µm.

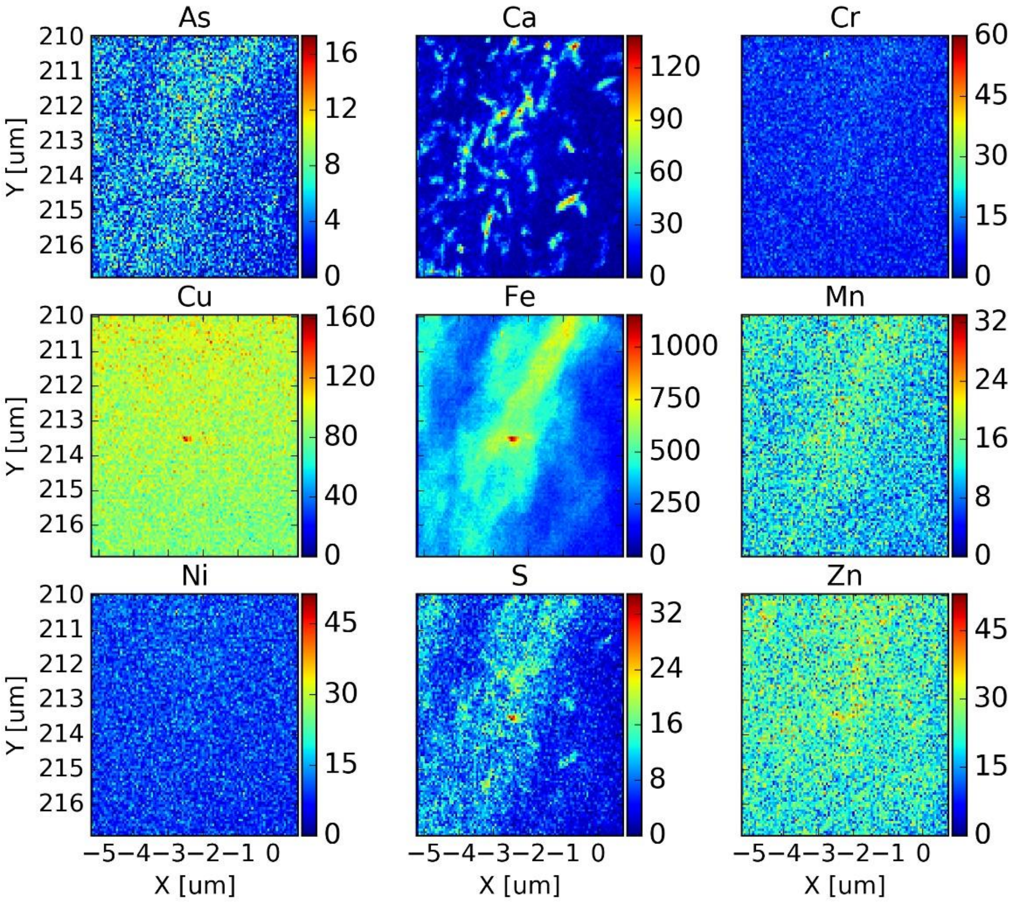

Figure S3. High resolution images (pixel size = 60 nm) from synchrotron X-ray nanofluorescence (nano-XRF) measurement showing distributions of As, Ca, Cr, Cu, Fe, Mn, Ni, S and Zn in the outer endodermis of *Spartina alterniflora* root (Area S2505). This nanometer scale mapping is able to provide more accurate information of the elemental distributions in the tissue than that from the micrometer scale measurement and, hence, reduce the uncertainty. The color bars show the concentrations in the units of counts per second (cps). The dimension of the area is approximately 6 μm × 6 μm.

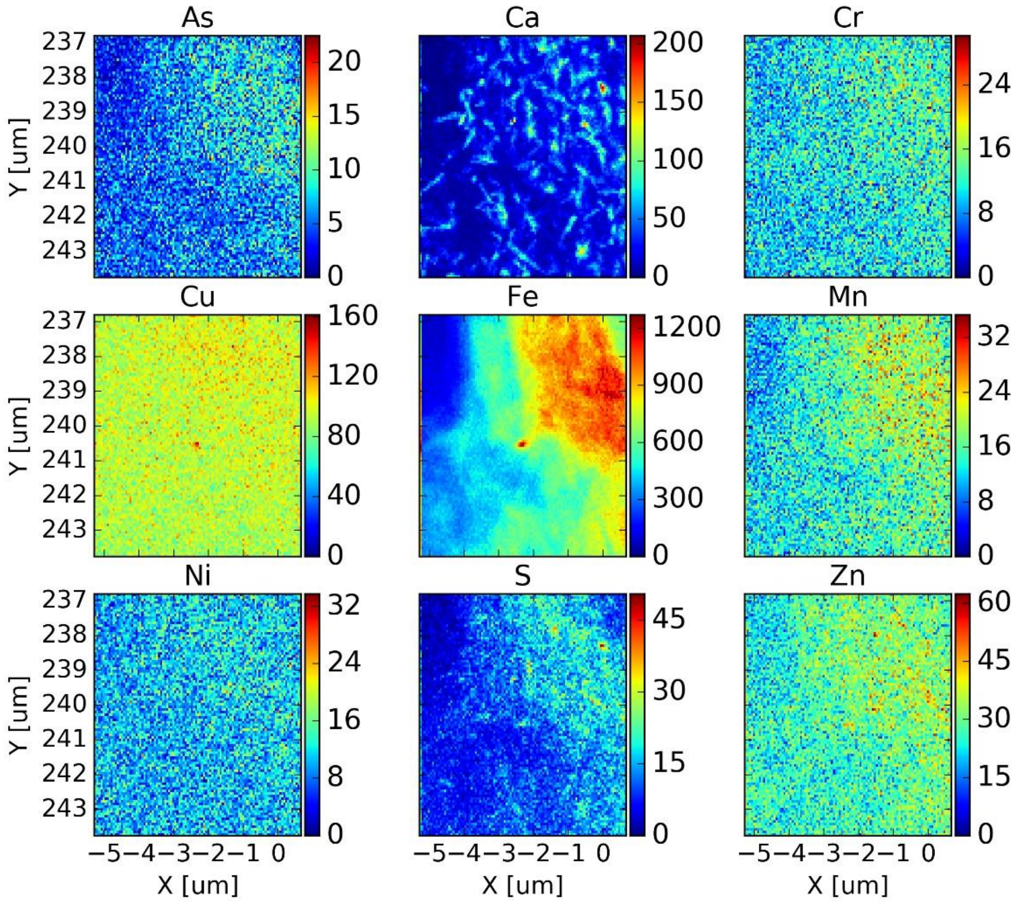

Figure S4. High resolution images (pixel size = 60 nm) from synchrotron X-ray nanofluorescence (nano-XRF) measurement showing distributions of As, Ca, Cr, Cu, Fe, Mn, Ni, S and Zn in the outer endodermis of *Spartina alterniflora* root (Area S2518). This nanometer scale mapping is able to provide more accurate information of the elemental distributions in the tissue than that from the micrometer scale measurement and, hence, reduce the uncertainty. The color bars show the concentrations in the units of counts per second (cps). The dimension of the area is approximately 6 μm × 6 μm.

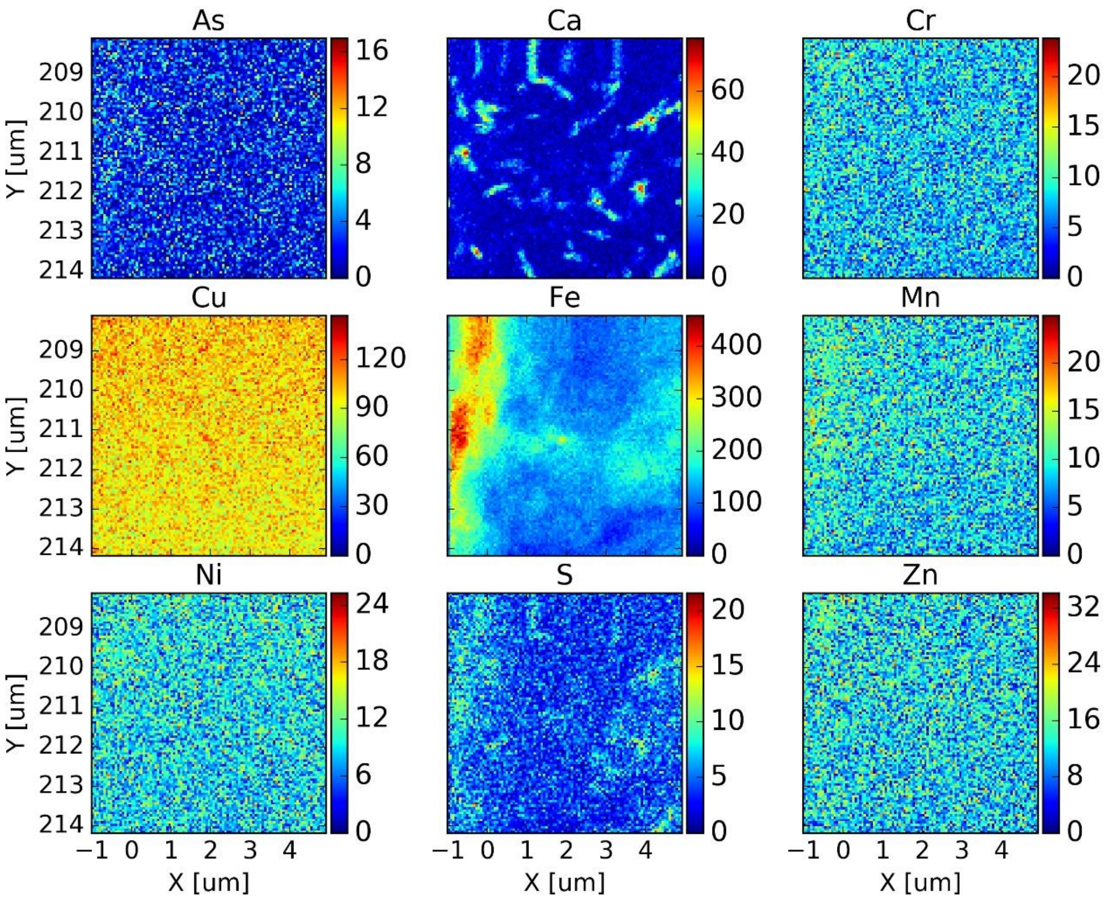

Figure S5. High resolution images (pixel size = 60 nm) from synchrotron X-ray nanofluorescence (nano-XRF) measurement showing distributions of As, Ca, Cr, Cu, Fe, Mn, Ni, S and Zn in the inner endodermis of the *Spartina alterniflora* root (Area S2504). This nanometer scale mapping is able to provide more accurate information of the elemental distributions in the tissue than that from the micrometer scale measurement and, hence, reduce the uncertainty. The color bars show the concentrations in the units of counts per second (cps). The dimension of the area is approximately 6 µm × 6 µm.

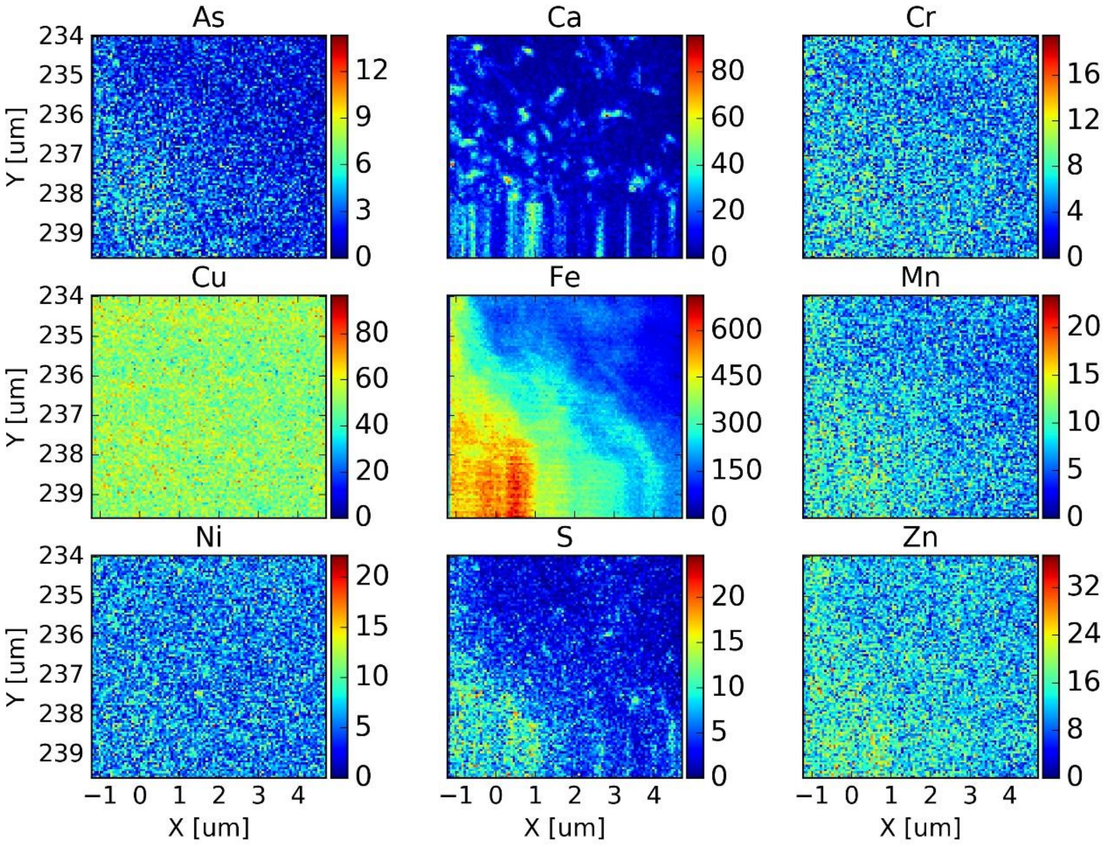

Figure S6. High resolution images (pixel size = 60 nm) from synchrotron X-ray nanofluorescence (nano-XRF) measurement showing distributions of As, Ca, Cr, Cu, Fe, Mn, Ni, S and Zn in the inner endodermis of the *Spartina alterniflora* root (Area S2516). This nanometer scale mapping is able to provide more accurate information of the elemental distributions in the tissue than that from the micrometer scale measurement and, hence, reduce the uncertainty. The color bars show the concentrations in the units of counts per second (cps). The dimension of the area is approximately 6 μm × 6 μm.
